# Rapid oxidative release of fungal mannan for detection by immunoassay

**DOI:** 10.1093/mmy/myac066

**Published:** 2022-09-06

**Authors:** Alexander J Kvam, Amanda R Burnham-Marusich, Michael Mash, Thomas R Kozel

**Affiliations:** Department of Microbiology and Immunology, University of Nevada, Reno School of Medicine, Reno, NV, 89557, USA; DxDiscovery, Inc., Reno, NV, 89557, USA; DxDiscovery, Inc., Reno, NV, 89557, USA; Department of Microbiology and Immunology, University of Nevada, Reno School of Medicine, Reno, NV, 89557, USA; DxDiscovery, Inc., Reno, NV, 89557, USA

**Keywords:** mannan, immunoassay, oxidative release, point-of-care, dermatophyte

## Abstract

Detection of fungal cells in infected tissue by procedures such as potassium hydroxide (KOH) microscopy and histopathology are well-established methods in medical mycology. However, microscopy requires skilled personnel, specialized equipment, and may take considerable time to a result. An alternative approach is immunoassay for detection of fungal mannans in tissue as a biomarker for the presence of fungal cells. However, mannan is a component of the fungal cell wall, and detection of mannan would require a facile means for mannan extraction prior to detection by immunoassay. In this study, we evaluated a broad spectrum of extraction reagents using *Trichophyton rubrum* mycelia and *Saccharomyces cerevisiae* Mnn2 blastoconidia as model fungi. Oxidative release by treatment with dilute bleach proved to be a novel and highly effective procedure. Complete extraction occurred in as little as 2-4 min. Detergents, chaotropes, and acid were ineffective. Strong base released mannan but was less efficient than oxidative release and required the use of highly corrosive reagents. Oxidative release of cell wall mannans from fungal mycelia and blastoconidia may be an effective first step in immunodetection of fungi in tissues from infected humans, animals, or plants that could be done at or near the diagnostic point of need.

## Introduction

The cell wall of most fungal species has an inner core of branched β-1,3 and β-1,6 glucan that is linked to chitin via a β-1,4 linkage.^1^ The outer layers of the fungal cell wall include highly mannosylated glycoproteins. Most mannoproteins of the Ascomycota and Mucorales have an α-1,6 mannose backbone.^2,3^ The proteins are modified with *N*-linked and *O*-linked mannans. *O*-linked mannans have shorter chains of 4-5 mannose residues, whereas *N*-linked mannans have a long outer chain of up to 200 mannose residues with an α-1,6-linked mannose backbone.^4,5^ Fungal mannoproteins are decorated with sometimes complex side chains that typically include mannose in the case of *Saccharomyces*^6^ and *Candida*,^7^ galactose in the case of pathogens such as *Aspergillus*,^8^*Histoplasma*^9,10^ and *Blastomyces*,^11^ and fucose in the case of many Mucorales.^12,13^

Fungal cell wall mannans are shed into body fluids such as blood and urine during invasive fungal disease. Indeed, shed mannans have been recognized as biomarkers for every invasive infection produced by an ascomycetous fungus that has been studied to date. Examples include i) galactomannans (Gm) found in serum or urine during aspergillosis,^14,15^ histoplasmosis,^9,10^ blastomycosis,^11^ coccidioidomycosis,^16^ and fusariosis,^17,18^ and ii) mannans (Mn) with invasive candidiasis.^19–21^ In each case, free mannan was detected by immunoassay using monoclonal or polyclonal antibodies as detection reagents.

Many fungal infections are localized in tissues without systemic spread. Examples include dermatophyte infection, vaginal candidiasis, fungal keratitis, and rhino-orbital-cerebral mucormycosis. The presence of fungi in tissue is determined by culture, KOH microscopy, or histopathology. These traditional approaches are widely accepted, but may require considerable time to a result, trained personnel, and laboratory infrastructure. An alternative approach would be extraction of cell wall mannan from infected tissue and detection of extracted mannan by immunoassay. There is considerable precedent for rapid immunoassay of extractable antigen for the detection of microbes in tissue. For example, acid extraction followed by neutralization and immunodetection of the cell wall polysaccharide of Group A Streptococcus is the basis for widely used point-of-care (POC) immunoassays for diagnosis of streptococcal pharyngitis.^22^

Historically, cell wall mannans have been extracted from fungi by use of the hot citrate method of Peat.^23^ Although effective, this procedure requires autoclaving of fungi, a procedure that is not well suited for field use or the POC. The goal of this study was to identify procedures for extraction of mannans for subsequent analysis by immunoassay that can be done rapidly at the point of diagnostic need. Our results found that treatments of fungi with alkali or bleach are effective extraction procedures. Of these procedures, oxidative release of mannans is best suited for rapid POC use.

Two fungi were chosen as model microbes for mannan extraction. Mycelia from *Trichophyton rubrum* were used as a representative example of a dermatophyte. We also wanted to determine whether any extraction procedure could have broad applicability across fungi. To this end, cells of *Saccharomyces cerevisiae* Mnn2 were chosen for study because mAb 2DA6, which was used to construct the mannan immunoassay, is specific for the α-1,6 mannose found in the backbone of fungi of the Zygomycetes and Ascomycetes.^3^ Mnn2 is an *S. cerevisiae* mutant that lacks side chains and is composed only of the α-1,6 mannose backbone.^24,25^ As a consequence, mAb 2DA6 is highly reactive with Mnn2.^3^

## Materials and Methods

### Fungal Cultures

*Trichophyton rubrum* was purchased from ATCC (ATCC 4438). *Trichophyton rubrum* was grown in liquid RPMI 1640 (Caisson Laboratories) with 2% glucose for 14 days at 25°C on a rotary shaker. *Saccharomyces cerevisiae* Mnn2 mutant (clone ID 33152) was obtained from GE Dharmacon. Yeast was grown in liquid RPMI 1640 with 2% glucose and 40 μg/ml uridine (Sigma-Aldrich) for 48 h at 30°C on a rotary shaker.

### Preparation of cells for mannan extraction

*Trichophyton rubrum* cells were separated from the growth medium with a 0.45 μm filter (VWR). Cells were washed four times with purified water. Cells of *S. cerevisiae* Mnn2 were similarly washed with water over a 0.2 μm filter. All cells were transferred to 50 ml tubes, washed once with 70% ethanol, once with 85% ethanol, once with 95% ethanol, and three times with 100% ethanol. Cells were then washed three times with acetone. The acetone-treated cells were dried for two days at room temperature. A portion of the dried cells was cultured to confirm the lack of viability. Dried *T. rubrum* mycelia were weighed, resuspended in sterile water, ground lightly to achieve a homogenous suspension at 1 mg/ml (Fisher disposable tissue grinder, 302-542-09), and aliquoted into individual tubes at 100 μg/aliquot. The dried Mnn2 cells were similarly processed, but did not require grinding to remove clumps. Aliquots of fungal cells were frozen at −20ºC until use.

### Mannan extraction

Various chemicals were tested for the ability to extract mannan from cell walls of *T. rubrum* and Mnn2. Mycelia of *T. rubrum* and Mnn2 yeast cells from frozen aliquots were collected by centrifugation (2500 × g for 5 min). The supernatant fluid was discarded, and 300 μl of extraction reagent was added to the cell pellets followed by vortexing. All extractions were performed at room temperature. After extraction reagent was added, extraction was allowed to proceed for 30 min. The extraction time was varied for time-course experiments. Extractions were stopped using two different methods: for treatment using detergents and chaotropes, fungal cells were removed from the extraction buffer by centrifugation. The supernatant was collected for immunoassay testing. For extraction with acid, base or oxidizing reagents, extraction was stopped by adding an appropriate amount of neutralization reagent and clarified by centrifugation. The supernatant fluid was collected for immunoassay testing.

### Extraction reagents

All reagent solutions were made in 18 Mohm-cm H_2_O. The detergents sodium dodecyl sulfate (SDS) (Fisher Scientific, BP166-500), 3-[(3-Cholamidopropyl) dimethylammonio]-1-propanesulfonate (CHAPS Hydrate) (Sigma-Aldrich, C3023-5 G), hexadecyltrimethylammonium bromide (CTAB) (Sigma Aldrich, H9515-100 G) and sodium deoxycholate (Sigma-Aldrich, D6750) were each tested at various wt/vol ratios. Tween 20 (VWR Life Sciences, M147) was tested on a (vol/vol) basis. The chaotropes lithium acetate (Acros Organics, 297 110 250) and sodium thiocyanate (Sigma-Aldrich, 251 410) were each tested on the basis of molarity. The oxidizers sodium hypochlorite (NaClO), hydrogen peroxide and peracetic acid were each evaluated as vol/vol. Extraction with the bases sodium hydroxide (Macron, 7708-06) and trisodium phosphate (Macron, 7940-04) and the acids hydrochloric acid (Acros Organics, 42379-5000) and sulfuric acid (BDH, 3072) were done on the basis of molarity.

Supernatant fluids from NaClO-extracted cells were neutralized with a 2-molar excess of sodium thiosulfate pentahydrate (Acros Organics, 42446-0010). Supernatant fluids from base- or acid-treated cells were neutralized with an excess of PBS, pH 7.4 (274 m m NaCl, 5.37 m m KCl, 20.3 m m Na_2_HPO_4_, 3.5 m m KH_2_PO_4_).

### Detection of mannan by ELISA

An antigen-capture ELISA in sandwich format was used to assess the relative amounts of mannan in extracts from treated cells. Details of the ELISA have been described.^3^ Briefly, all assays were done using microtiter plates that were coated with mAb 2DA6 that captured the mannan from the solution. The plates were incubated with serial dilutions of mannan extract, washed, and incubated with horseradish peroxidase-labeled mAb 2DA6, washed, and incubated with the substrate. mAb 2DA6 is reactive with the α-1,6 mannan backbone of the cell wall mannan of the Zygomycetes and Ascomycetes.^3^ Results from the mannan immunoassay are reported as the reciprocal of the endpoint dilution of extract that produced an OD_450_ of 0.5 using a log-log plot.

In all cases, results are shown for one of two similar experiments with similar results.

## Results

### Efficiency of various treatments for extraction of mannan

Mycelia of *T. rubrum* were treated for 30 min with i) detergents, ii) chaotropes, iii) acid, iv) base, and v) oxidizers. The titers of supernatant fluids from the various extraction procedures were compared with levels of mannan from mycelia extracted by use of hot citrate as described by Peat.^23^ The hot citrate extraction provided a positive control for the amount of mannan that can be extracted using a conventional procedure. Mycelia were incubated with water as a negative control.

**Figure 1.**
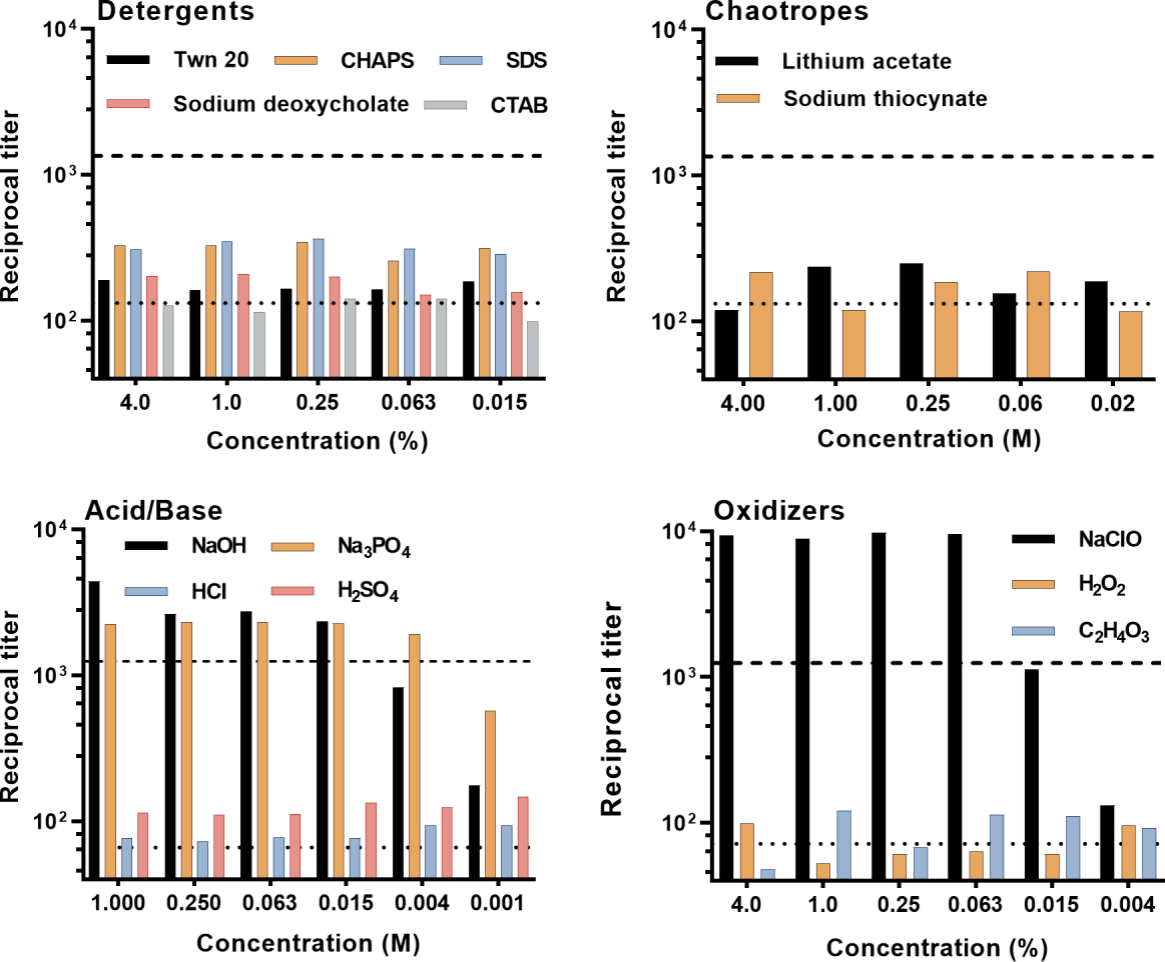
Extraction of mannans from mycelia of *T. rubrum* by treatment for 30 min with detergents, chaotropes, acid, base, and oxidizers. Results are reported as the reciprocal titer of supernatant fluid in an antigen-capture ELISA. Upper dashed line – extraction by treatment with hot citrate (positive control). Lower dotted line – titer after treatment with water alone (negative control).

Treatment of mycelia with the detergents Tween 20, CHAPS, SDS, sodium deoxycholate, and CTAB extracted little or no mannan (Fig. 1). Similarly, the chaotropes lithium acetate and sodium thiocyanate and the acids HCl and H_2_SO_4_ had no ability to extract mannan from mycelia. In contrast, treatment with NaOH and Na_3_PO_4_ were effective extraction reagents over the range of 0.015-1.0 M. Finally, the oxidant NaClO proved highly effective at extraction over the range of 0.063-4% vol/vol. Indeed, the amount of mannan extracted with NaClO was almost 10 times greater than what was extracted via the hot citrate method. Two other oxidants, H_2_O_2_ and peracetic acid, did not extract mannan from *T. rubrum* mycelia.

**Figure 2.**
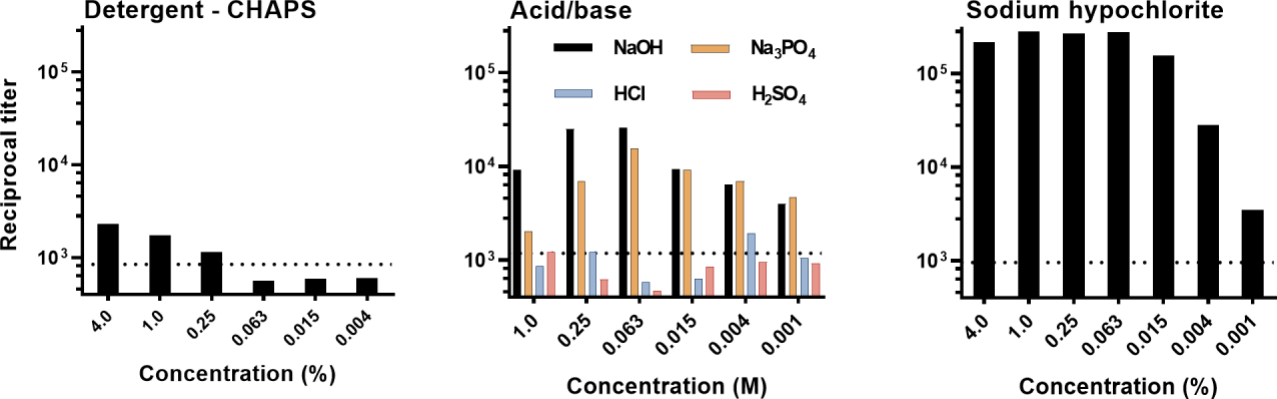
Extraction of mannans from yeast cells of *S. cerevisiae* Mnn2 by treatment for 30 min with detergent (CHAPS), acid, base, and the oxidizer, sodium hypochlorite. Results are reported as the reciprocal titer of supernatant fluid in an antigen-capture ELISA. Dotted line – titer after treatment with water alone (negative control).

Complementary experiments were performed with *S. cerevisiae* Mnn2 with selected extraction reagents to determine if the pattern of mannan extraction observed with *T. rubrum* was also found with a distantly related fungus, *S. cerevisiae* (Fig. 2). Treatment with the detergent CHAPS showed little extraction of mannan. As with *T. rubrum*, mannan was extracted by treatment with NaOH and Na_3_PO_4_ but not with HCl or H_2_SO_4_. Finally, NaClO was highly effective in extracting mannan from *S. cerevisiae* Mnn2.

### Effect of treatment time on extraction with NaOH and NaClO

**Figure 3.**
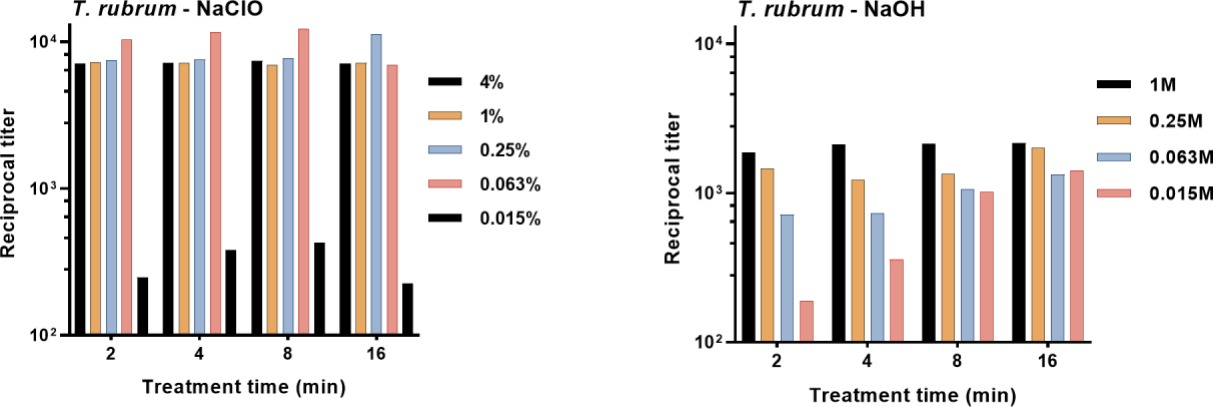
Impact of treatment time and concentration on extraction of mannan from mycelia of *T. rubrum* by treatment with sodium hypochlorite and sodium hydroxide. Results are reported as the reciprocal titer of supernatant fluid in an antigen-capture ELISA.

Results in Figs. 1 and 2 showed the relative amounts of mannan that were extracted by treatment for 30 min. An immunoassay that would be suitable for diagnosis of fungal infection at the point of care would require extraction after a shorter treatment time. To this end, cells of *T. rubrum* were treated with various concentrations of NaOH or NaClO for 2–16 min. The extracted mannan was then evaluated by mAb 2DA6 antigen-capture ELISA. The results (Fig. 3) showed that treatment with 0.063-4% NaClO produced complete extraction across the 2-16 min treatment times. In contrast, variation in treatment time and reagent concentration considerably impacted the extent of mannan extraction by NaOH.

**Figure 4.**
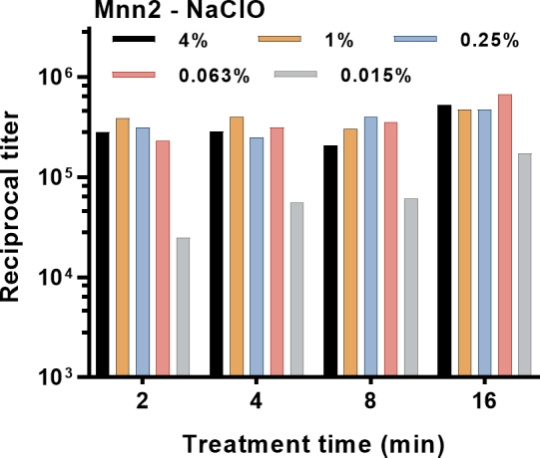
Impact of treatment time and concentration on extraction of mannan from *S. cerevisiae* Mnn2 cells by treatment with NaClO. Results are reported as the reciprocal titer of supernatant fluid in an antigen-capture ELISA.

Given the potent ability of NaClO as a mannan extraction reagent with *T. rubrum*, we confirmed the results by a determination of the effect of NaClO concentration and treatment time in extraction of mannan from *S. cerevisiae* Mnn2. The results were identical to those obtained with *T. rubrum*; treatment with 0.063-4% NaClO produced complete extraction over the 2-16 min treatment times (Fig. 4).

## Discussion

Immunoassay for circulating fungal mannans is a useful aid to diagnose a variety of invasive fungal infections including aspergillosis, candidiasis, and the endemic fungi.^9–11,14–21^ In these infections, fungal elements are found in multiple tissues, and mannan is shed into the circulation and can be detected in blood or urine. The goal of the present study was to identify means for rapid and efficient extraction of mannans from intact fungi. Such procedures would be critical to point-of-care immunoassays that could be used to directly detect fungi in clinical specimens from patients with suspected onychomycosis, vaginal candidiasis, fungal keratitis, or rhino-ocular cerebral mucormycosis. Indeed, immunoassay for extracted mannan could be a surrogate for the classical KOH microscopy used to identify mycelia in tissue. Toward this goal, we evaluated means for extraction of mannans from the dermatophyte *T. rubrum* and the Mnn2 mutant of *S. cerevisiae*. Both were selected as model fungi due to the high reactivity of their mannans with mAb 2DA6, which was used for construction of the antigen-capture ELISA used in this study for detection of extracted mannans.^3^

Two treatments proved particularly effective at extraction of mannan from the target fungi: alkali and NaClO, household bleach. Alkali soluble fractions of cell wall polysaccharides have been recognized for many years (reviewed in^26^). For example, NaOH was used to extract galactomannans from whole cells of *H. capsulatum, P. brasiliensis* and *B. dermatitidis*.^27^ Oxidative release of natural glycans was recently described as a means for preparation of *N*- and *O*-glucans from natural glycoproteins.^28,29^ NaClO selectively degraded the aglycon portion of native glycoconjugates to release intact glycans. Our results extend the spectrum of glycoproteins susceptible to oxidative release of natural glycans to include fungal mannoproteins. Oxidative release of mannans also has the potential to facilitate structural analysis of fungal mannans because the treatment yields glycans that are free of protein.^28^

Despite the effectiveness of NaOH as an extraction reagent, treatment with alkali would have limited value for POC use because the extraction required high concentrations of caustic extraction and neutralization reagents. In contrast, oxidative release of mannans had many features needed for POC specimen treatment. Oxidative release from fungal cells was rapid, providing complete release over a 2-8 min time frame. Moreover, mannan extraction was accomplished by concentrations of NaClO as low as 0.06-1%, well below the 5-6% concentration in household bleach. As a consequence, the process does not involve use of highly toxic chemicals. Finally, neutralization of NaClO was readily accomplished by use of sodium thiosulfate, which is also a common household and industrial bleach neutralizer. Sodium thiosulfate has low toxicity and is a classical antidote to cyanide poisoning.^30^

There is an unmet need in the clinic for rapid, POC diagnosis of fungal infections. Consequently, methods for POC mannan extraction followed by immunoassay for diagnosis of fungal infection would be a valuable tool for public health and are an area of high interest. Noriki and Ishida recently described an immunoassay for detection of dermatophytes.^31^ In this assay, an antigen was extracted from dermatophytes using non-ionic or zwitterionic surfactants^32^ and detected by use of mAb 0014.^31^ The antigen detected by mAb 0014 was determined to be a polysaccharide on the basis of susceptibility to degradation by periodate and resistance to degradation by protease.^31^ Results of the present study, which showed a failure to extract the α-1,6 mannan detected by mAb 2DA6 with either non-ionic or zwitterionic detergents (Fig. 1), differentiate the mannan biomarker of the present study from the detergent-extractable carbohydrate described by Noriki.^32^ An additional difference between the specificity of mAb 2DA6 of the present study and mAb 0014 is the positive reactivity of mAb 0014 with fixed mycelia of *Fusarium oxysporum*.^31^ and the exceedingly poor reactivity of mAb 2DA6 with mannan extracted from *Fusarium* spp.^3^ To our knowledge, the present study represents the first systemic experimental analysis of multiple classes of chemical reagents for their efficiency at extracting mannan from fungi and for their compatibility with downstream immunoassay detection.

Finally, a caveat regarding the results should be noted. All results are based on immunoassay using reactivity with mAb 2DA6 as an indicator for mannan extraction. Our results do not indicate whether any of the treatments may have altered the mannan or destroyed the epitope recognized by mAb 2DA6. This caveat does not alter our conclusion that base and NaClO are effective reagents for extraction of mannan that is reactive in an immunoassay.

Oxidative release of mannans from intact fungal cells is a promising technology for identification of the presence of fungal cells in specimens from human, animal, or plant fungal infections. Our results showed that mannans released by treatment with NaClO and then neutralized with sodium thiosulfate were readily detected in an immunoassay using the model fungi, *T. rubrum* and *S. cerevisiae* Mnn2. The process was rapid, did not require toxic or caustic reagents, and released mannans that were readily detected by mannan-specific immunoassay. These results provide proof of concept for a potential POC assay for the detection of mycelia in tissue. The next step will be the translation of this technology into a point-of-care lateral flow immunoassay diagnostic that combines extraction and detection in a sample-to-answer assay using clinical specimens. This approach would be analogous to the well-established POC antigen-detection tests for Group A streptococcal pharyngitis which use acid for extraction of bacterial polysaccharides.^33^ Future directions for this technology include the release of mannans from patient specimens with fungal infections where KOH microscopy is currently used for diagnosis of fungal infection, e.g., onychomycosis, fungal keratitis, or *Candida* vaginitis. To this end, ongoing studies in progress aim to produce mAbs that are specific for mannans of the dermatophytes, various species of *Candida, Aspergillus*, and members of the Mucorales. A critical component of these ongoing studies is a determination of matrix effects on mannan extraction. The goal is a library of diagnostic tests that are sensitive, specific, and rapid for the diagnosis of fungal infection.
